# Differences in Care Team Response to Patient Portal Messages by Patient Race and Ethnicity

**DOI:** 10.1001/jamanetworkopen.2024.2618

**Published:** 2024-03-18

**Authors:** Mitchell Tang, Rebecca G. Mishuris, Lily Payvandi, Ariel D. Stern

**Affiliations:** 1 Harvard Graduate School of Arts and Sciences, Cambridge, Massachusetts; 2 Harvard Business School, Boston, Massachusetts; 3 Digital, Mass General Brigham, Somerville, Massachusetts; 4 Department of Medicine, Brigham and Women’s Hospital, Boston, Massachusetts; 5 Department of Family Medicine, Boston Medical Center, Boston, Massachusetts; 6 Boston University School of Medicine, Boston, Massachusetts; 7 Harvard-MIT Center for Regulatory Science, Boston, Massachusetts

## Abstract

**Importance:**

The COVID-19 pandemic was associated with substantial growth in patient portal messaging. Higher message volumes have largely persisted, reflecting a new normal. Prior work has documented lower message use by patients who belong to minoritized racial and ethnic groups, but research has not examined differences in care team response to messages. Both have substantial ramifications on resource allocation and care access under a new care paradigm with portal messaging as a central channel for patient–care team communication.

**Objective:**

To examine differences in how care teams respond to patient portal messages sent by patients from different racial and ethnic groups.

**Design, Setting, and Participants:**

In a cross-sectional design in a large safety-net health system, response outcomes from medical advice message threads sent from January 1, 2021, through November 24, 2021, from Asian, Black, Hispanic, and White patients were compared, controlling for patient and message thread characteristics. Asian, Black, Hispanic, and White patients with 1 or more adult primary care visits at Boston Medical Center in calendar year 2020 were included. Data analysis was conducted from June 23, 2022, through December 21, 2023.

**Exposure:**

Patient race and ethnicity.

**Main Outcomes and Measures:**

Rates at which medical advice request messages were responded to by care teams and the types of health care professionals that responded.

**Results:**

A total of 39 043 patients were included in the sample: 2006 were Asian, 21 600 were Black, 7185 were Hispanic, and 8252 were White. A total of 22 744 (58.3%) patients were women and mean (SD) age was 50.4 (16.7) years. In 2021, these patients initiated 57 704 medical advice request message threads. When patients who belong to minoritized racial and ethnic groups sent these messages, the likelihood of receiving any care team response was similar, but the types of health care professionals that responded differed. Black patients were 3.95 percentage points (pp) less likely (95% CI, −5.34 to −2.57 pp; *P* < .001) to receive a response from an attending physician, and 3.01 pp more likely (95% CI, 1.76-4.27 pp; *P* < .001) to receive a response from a registered nurse, corresponding to a 17.4% lower attending response rate. Similar, but smaller, differences were observed for Asian and Hispanic patients.

**Conclusions and Relevance:**

The findings of this study suggest lower prioritization of patients who belong to minoritized racial and ethnic groups during triaging. Understanding and addressing these disparities will be important for improving care equity and informing health care delivery support algorithms.

## Key Points

**Question:**

Amidst increased patient portal messaging, are there differences in care team response to messages from Asian, Black, and Hispanic patients compared with similar White patients?

**Findings:**

In this cross-sectional study including 39 043 patients, medical advice request messages sent by patients who belong to minoritized racial and ethnic groups were less likely to receive a response from attending physicians and more likely to receive a response from registered nurses, suggesting lower prioritization during triaging. The differences observed were similar among Asian, Black, and Hispanic patients.

**Meaning:**

The findings of this study suggest potential disparities in how health care team resources are allocated across portal messages by patient race and ethnicity; understanding and addressing these disparities will be necessary for improving care equity.

## Teaser

This cross-sectional study assesses differences in care team response to patient portal messages by patient race and ethnicity among primary care patients in the US.

## Introduction

Patient portals are defined by the Office of the National Coordinator for Health Information Technology as “secure online websites that give patients convenient 24-hour access to personal health information.”^1^ Since the passage of the Health Information Technology for Economic and Clinical Health Act in 2009, patient portals have become much more commonplace.^2^ More recent restrictions on information blocking by health care institutions with the 21st Century Cures Act have increased the breadth of information available to patients via patient portals.^3^ Nevertheless, only 38% of patients surveyed in the first quarter of 2020 said they had accessed a patient portal in the past 12 months.^4^

At the onset of the COVID-19 pandemic, patient portal use increased substantially, most notably in the form of secure messaging between patients and their health care teams.^5^ Higher message volumes have largely persisted, reflecting a new normal, with portal messaging as a central channel for patient–health care professional interaction.^6^

Portal messages can increase patients’ access to care teams but can also place substantial burdens on health care professionals.^7,8^ Amidst this surge in use, message triaging has become an important tool to manage physician burden. Most practices use centralized message pools where triaging nurses decide to either respond to messages themselves or forward them to another health care professional, including escalating messages to an attending physician.

Prior work has shown that Black and Hispanic patients are less likely to send messages, raising concerns that, as increasing resources are allocated to responding to portal messages, care disparities may become further entrenched.^9^ However, to our knowledge, no prior work has examined differences in how care teams respond to patients’ messages.

A large body of research has highlighted racial bias within health care, with many health care professionals having implicit negative biases toward patients who belong to minoritized racial and ethnic groups.^10^ National surveys have shown that nearly 20% of patients report experiencing racial discrimination in a health care interaction, with rates as high as 50% for Black patients.^11^ Recent work has also reported higher rates of stigmatizing language in the electronic health records (EHRs) of Black patients.^12,13^ Within the context of portal messaging, racial bias may manifest in lower likelihood of care team response for messages from patients who belong to minoritized racial and ethnic groups. With nurse triaging, there is the specific concern that messages from patients who belong to minoritized racial and ethnic groups are less likely to be prioritized for physician response. Patient health literacy may also play an important role. Prior work has observed that patients who belong to minoritized racial and ethnic groups often have lower health literacy, even after controlling for factors such as educational level, income, gender, and age.^14^ Lower health literacy may influence the types of requests patients make through the portal and the manner in which those requests are communicated. Both may impact care team response.

In our study we examined differences in care team response to patient portal messages by patient race and ethnicity among primary care patients at Boston Medical Center (BMC). We focus our analysis on medical advice request messages, which are the main form of patient-initiated portal message at BMC. These messages enable patients to make short text-based communications to their care teams, such as asking clarifying questions about their postvisit instructions, updating care teams on their symptom trajectory, or asking about new symptoms. They may also be used for more logistical communications, such as scheduling requests. We examined whether patient messages receive a care team response and the types of care team members who respond. Both findings may have ramifications for health equity and the allocation of resources under the new care paradigm in which patient portals constitute an increasingly central channel for patient–care team interaction. Growing interest in artificial intelligence (AI) tools for triaging and responding to patient messages^15,16^ heightens the importance of understanding these potential disparities, given the propensity of AI to perpetuate underlying inequalities in the data upon which algorithms were trained.^17,18^

## Methods

The institutional review boards from BMC and Harvard University ruled this study exempt because it was a secondary analysis of routinely collected data and posed minimal risk to participants. We followed the Strengthening the Reporting of Observational Studies in Epidemiology (STROBE) reporting guideline for cross-sectional studies.

### Setting

Our study sample included patients with at least 1 adult primary care visit at BMC in calendar year 2020 (primary care active in 2020). Boston Medical Center is the largest safety-net health care organization in New England, mainly serving residents of Boston and surrounding towns. The BMC health system includes primary care and specialty outpatient clinics, emergency departments (EDs), and inpatient hospitals. At BMC, 50% of patients have incomes at or below the federal poverty level, 30% speak a primary language other than English, and 80% of care is reimbursed through a government payer.^19^ Boston Medical Center is a uniquely compelling setting for a study of disparities across race and ethnicity; while the BMC patient population is racially and ethnically diverse, it is relatively homogeneous socioeconomically. Thus, many socioeconomic factors that race and ethnicity might otherwise be proxies for, such as educational attainment, income, or insurance status, are not associated with race and ethnicity to the same degree in our setting.

### Data Sources

For each patient in our sample, we extracted data from the BMC Epic (Epic Systems Corporation) EHR on all patient-initiated medical advice request messages sent from January 1 through November 24, 2021, and all associated care team responses. Messages in the same response chain were linked into message threads. For each message, we observed the date and time the message was sent, the recipient department, and whether it had an attachment. Message text was not available for the purpose of this study. Each thread could also be linked to the portal session in which the initial message was sent. For each of those portal sessions, we observed whether the session was conducted via the Epic MyChart mobile app or through a web browser, and which type of mobile device was used to access the mobile app (Android or iPhone). Additionally, we observed which other portal functionalities were accessed during the portal sessions (eg, medications, test result details, and clinical notes).

We also extracted data on all outpatient visits, ED visits, and inpatient stays from January 1, 2020, through November 24, 2021, for all patients in our sample. Patient demographic data, including self-reported race and ethnicity, sex, age, zip code of residence, health insurer, and preferred language, reflected patients’ latest record in the EHR as of November 24, 2021. Race and ethnicity data were collapsed into a single variable. All patients of Hispanic or Latino ethnicity were grouped (hereafter referred to as Hispanic), regardless of race. The remaining non-Hispanic patients were categorized by race. Our analyses focused on non-Hispanic Asian patients (Asian), non-Hispanic Black patients (Black), Hispanic patients, and non-Hispanic White patients (White). The remaining race and ethnicity groups tracked in the EHR data were American Indian or Alaska Native, Native Hawaiian or Other Pacific Islander, and a catch-all Other category. However, these groups lacked sufficient sample size for statistical analysis in our data set, and we opted to exclude them rather than group them, given an expected high degree of heterogeneity among patients from these groups. We also excluded individuals with unknown race and ethnicity.

Patient health status was coded with binary indicators for 27 common conditions based on the Chronic Conditions Data Warehouse (eTable 1 in Supplement 1).^20^ These indicators were populated based on patients’ EHR records, including their outpatient encounter diagnoses, diagnoses on their problem list, and primary diagnoses from their inpatient encounters. Data from the 2022 American Community Survey provided information on the median household income in each patient’s zip code of residence.^21^

### Outcomes

For each medical advice request thread, we noted whether it received at least 1 reply from a BMC care team member. We also examined the types of care team members that responded. These categories included (1) attending physician, (2) registered nurse (RN), (3) advanced practice professional, (4) resident physician, and (5) other (including medical assistants and social workers). We created binary flags for each thread representing whether there was at least 1 response from each of these categories.

Additional secondary response outcomes included (1) the number of care team responses, (2) hours until first care team response, and (3) whether the thread ended on a patient message. We also included a set of outcomes capturing the type of care team member that gave the first care team response of the thread, using the same 5 categories.

### Statistical Analysis

Data analysis was conducted from June 23, 2022, through December 21, 2023. Data analysis was conducted in R, version 4.3.1 (R Project for Statistical Computing). Response outcomes were compared for message threads from Asian, Black, Hispanic, and White patients. Adjusted comparisons were conducted using ordinary least-squares regression. In our primary ordinary least-squares specification, results were adjusted for factors that might confound comparisons in response outcomes, including patient characteristics (sex, age, number of chronic conditions, and whether English was their preferred language) and message thread characteristics (the year and month, day of the week, and hour of the day the initial message was sent, as well as a fixed effect for the recipient department). Additional fixed effects were included for the patient’s health insurer, zip code of residence, and most seen primary care team member in 2020. Standard errors were clustered at the patient level. These regressions intended to capture differences in response outcomes for similar message threads sent to the same recipient department from comparable patients across the 4 focal patient race and ethnicity groups.

Sensitivity analyses were conducted examining the robustness of our results within specific message thread subgroups, such as threads directed at primary care departments or those from patients in specific age groups, from patients with specific chronic conditions, from patients with English as a stated preferred language, from patients with specific numbers of primary care office visits in the period leading up to the message, or from individuals with a hospitalization in the prior year. Additional sensitivity analyses examined message threads from patients who were primary care active in 2019 (rather than 2020) to avoid overlap between our patient sample qualification period and early disruptions from the pandemic, and analyses incorporating random effects rather than fixed effects to adjust for the threads’ recipient department and patients’ health insurer, zip code of residence, and most seen primary care professional in 2020.

Additionally, we examined the robustness of our results to other adjustments that further described characteristics of the initiating message (whether it was sent via mobile app or web, the type of device that accessed the mobile app, whether it included an attachment, and what portal functionalities were used in the same portal session as the initial message); the patient’s number of outpatient, ED, and inpatient encounters immediately before and after the focal message; patients’ 2020 experience with medical advice messaging; and 2020 outpatient visit volume within the recipient department (additional details provided in the eMethods in Supplement 1).

## Results

### Sample Characteristics

A total of 42 395 patients were primary care active at BMC in 2020, of whom 39 043 were classified as being in 1 of our 4 in-scope race and ethnicity groups and were included in the analysis sample: 2006 were Asian, 21 600 were Black, 7185 were Hispanic, and 8252 were White. A total of 22 744 patients (58.3%) were women, 16 299 (41.7%) were men, and mean (SD) age was 50.4 (16.7) years. Compared with White patients, Black and Hispanic patients were more likely to be female, older, have a non-English preferred language, live in a zip code with a lower median household income, and have a higher chronic disease burden and level of care use at BMC (Table 1). These differences were somewhat attenuated across patients who sent a medical advice request message in 2021 (eTable 2 in Supplement 1). These differences motivated inclusion of several patient characteristic controls in our adjusted comparisons.

**Table 1.** Characteristics of Patients Who Were Primary Care Active at Boston Medical Center (BMC) in 2020

Characteristic^a^	Patient race and ethnicity group, No. (%)^b^				Total
	Asian	Black	Hispanic	White	
Total PCP active patients	2006 (5.1)	21 600 (55.3)	7185 (18.4)	8252 (21.1)	39 043 (100)
Sex					
Female	1139 (56.8)	13 105 (60.7)	4141 (57.6)	4359 (52.8)	22 744 (58.3)
Male	867 (43.2)	8495 (39.3)	3044 (42.4)	3893 (47.2)	16 299 (41.7)
Age, mean (SD), y	45.9 (17.2)	51.8 (16.5)	50.0 (16.7)	48.4 (16.9)	50.4 (16.7)
Age category, y					
18-24	86 (4.3)	596 (2.8)	271 (3.8)	232 (2.8)	1185 (3.0)
25-34	651 (32.5)	3514 (16.3)	1369 (19.1)	2162 (26.2)	7696 (19.7)
35-44	333 (16.6)	3721 (17.2)	1275 (17.7)	1522 (18.4)	6851 (17.5)
45-54	278 (13.9)	3922 (18.2)	1338 (18.6)	1089 (13.2)	6627 (17.0)
55-64	288 (14.4)	4533 (21.0)	1367 (19.0)	1542 (18.7)	7730 (19.8)
≥65	370 (18.4)	5314 (24.6)	1565 (21.8)	1705 (20.7)	8954 (22.9)
English preferred language	1344 (67.0)	17 255 (79.9)	3454 (48.1)	7833 (94.9)	29 886 (76.5)
No. of chronic conditions, mean (SD)	2.5 (2.9)	4.1 (3.3)	3.8 (3.3)	3.0 (3.1)	3.7 (3.3)
Patients with chronic conditions					
Hypertension	676 (33.7)	12 626 (58.5)	3515 (48.9)	3211 (38.9)	20 028 (51.3)
Diabetes	420 (20.9)	6765 (31.3)	2080 (28.9)	1141 (13.8)	10 406 (26.7)
Hyperlipidemia	691 (34.4)	8843 (40.9)	2818 (39.2)	2699 (32.7)	15 051 (38.5)
Depression	400 (19.9)	7610 (35.2)	3126 (43.5)	3206 (38.9)	14 342 (36.7)
Arthritis	319 (15.9)	6160 (28.5)	2039 (28.4)	2087 (25.3)	10 605 (27.2)
Anemia	392 (19.5)	8210 (38.0)	1944 (27.1)	1597 (19.4)	12 143 (31.1)
Chronic kidney disease	294 (14.7)	6352 (29.4)	1802 (25.1)	1308 (15.9)	9756 (25.0)
Cataracts	483 (24.1)	7704 (35.7)	2270 (31.6)	1532 (18.6)	11 989 (30.7)
Care use at BMC from January 1 to November 24, 2021, mean (SD),No.					
Primary care visits	3828 (1.91)	55 110 (2.55)	18 475 (2.57)	15 441 (1.87)	92 854 (2.38)
Non–primary care visits	13 163 (6.56)	187 426 (8.68)	63 837 (8.88)	68 824 (8.34)	333 250 (8.54)
Emergency department visits	358 (0.18)	10 424 (0.48)	3532 (0.49)	2246 (0.27)	16 560 (0.42)
Inpatient admissions	122 (0.06)	2872 (0.13)	777 (0.11)	742 (0.09)	4513 (0.12)
Zip code income quintile					
Highest	978 (48.8)	7281 (33.7)	2234 (31.1)	4702 (57.0)	15 195 (38.9)
Second	639 (31.9)	3765 (17.4)	1765 (24.6)	2129 (25.8)	8298 (21.3)
Third	261 (13.0)	5867 (27.2)	1472 (20.5)	832 (10.1)	8432 (21.6)
Fourth	63 (3.1)	829 (3.8)	425 (5.9)	257 (3.1)	1574 (4.0)
Lowest	55 (2.7)	3772 (17.5)	1269 (17.7)	265 (3.2)	5361 (13.7)

^a^ Patient age, zip code of residence, and health status reflected their latest record as of November 24, 2021 (end of our study period). Patients’ health status was represented through binary indicators for 27 common conditions (full list provided in eTable 1 in Supplement 1). These indicators were populated based on patients’ electronic health records, including their outpatient encounter diagnoses, diagnoses on their problem list, and primary diagnoses from their inpatient encounters. Zip code income quintiles are based on data from the 2022 American Community Survey^21^ and represent the median household income for patients’ zip code of residence; 0.5% of patients had missing zip code of residence data and were not included in the zip code income quintile measures.

^b^ Patient race and ethnicity groups were determined based on patient self-reported data recorded in the Boston Medical Center electronic health record. All patients of Hispanic or Latino ethnicity were grouped into the Hispanic group, regardless of race. The remaining non-Hispanic patients were categorized by race. Patients with a recorded race of American Indian or Alaska Native, Native Hawaiian or Other Pacific Islander, or Other were excluded due to insufficient sample size. Patients with unknown race and ethnicity were excluded as well.

### Unadjusted Comparisons Across Race and Ethnicity Groups

From January 1 through November 24, 2021, 10 686 analysis sample patients initiated 57 704 unique medical advice request message threads (eTable 2 in Supplement 1; Table 2). White patients had more than twice the mean messaging rate (2.8 threads per patient) compared with the other 3 race and ethnicity groups (Asian: 1.4, Black: 1.1, Hispanic: 1.2). Overall, 65.7% of threads received a care team response. Registered nurses and attending physicians were the 2 most common responding care team member types: 31.1% of threads received at least 1 RN response and 19.5% received an attending physician response (Table 2). Threads from White patients were slightly less likely to receive a care team response but were more likely to receive a response from an attending physician, especially compared with Black and Hispanic patients. Additional response outcomes are provided in eTable 3 in Supplement 1.

**Table 2.** 2021 Patient Medical Advice Request Message Thread Use and Care Team Response Outcomes for Patients Who Were Primary Care Active at Boston Medical Center in 2020^a^

Characteristic	Patient race and ethnicity group				
	Asian	Black	Hispanic	White	Total
Total PCP active patients, No. (% of total)	2006 (5.1)	21 600 (55.3)	7185 (18.4)	8252 (21.1)	39 043 (100)
Patient medical advice request threads, No. (% of total)	2714 (4.7)	23 174 (40.2)	8804 (15.3)	23 012 (39.9)	57 704 (100)
Patient medical advice request threads, mean (SD) per patient	1.4 (3.5)	1.1 (3.6)	1.2 (3.7)	2.8 (10.4)	1.5 (5.8)
Patient medical advice request threads with care team response, No. (% of threads sent by group)					
Any care team response	1923 (70.9)	15 372 (66.3)	5864 (66.6)	14 776 (64.2)	37 935 (65.7)
Any attending physician response	601 (22.1)	3861 (16.7)	1592 (18.1)	5220 (22.7)	11 274 (19.5)
Any registered nurse response	872 (32.1)	7897 (34.1)	2832 (32.2)	6335 (27.5)	17 936 (31.1)
Any advance practice professional response	150 (5.5)	1657 (7.2)	667 (7.6)	1646 (7.2)	4120 (7.1)
Any resident response	131 (4.8)	572 (2.5)	241 (2.7)	553 (2.4)	1497 (2.6)
Any other response	338 (12.5)	2901 (12.5)	1110 (12.6)	2576 (11.2)	6925 (12.0)

Abbreviation: PCP, primary care professional.

^a^ Use and response outcomes for medical advice request message threads sent from January 1 through November 24, 2021, by patients who were primary care active at Boston Medical Center in 2020 (at least 1 primary care outpatient visit in 2020). Medical advice request threads include a patient-initiated initial message and all other messages in the response chain.

### Adjusted Comparisons Across Race and Ethnicity Groups

**Figure 1.**
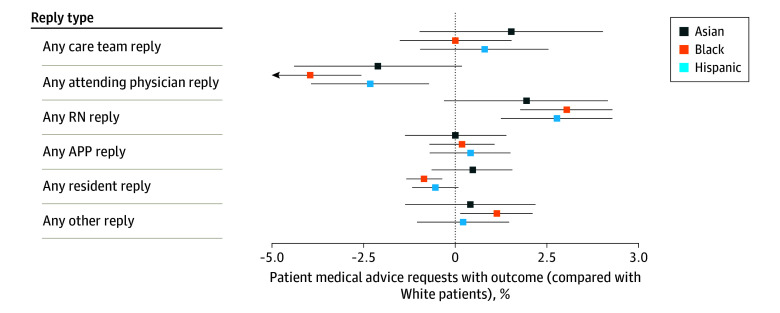
Care Team Response Rates for Patient Medical Advice Request Threads From Patients Who Were Primary Care Active at Boston Medical Center in 2020 Error bars reflect 95% CIs with heteroskedasticity-robust SEs clustered at the patient level. APP indicates advanced practice professional; RN, registered nurse.

After adjusting for patient and thread characteristics, we found relatively small differences in overall care team response rates across the 4 race and ethnicity groups in the analysis sample. However, we observed large differences in the types of care team members that replied. Black patients were 3.95 percentage points (pp) less likely (95% CI, −5.34 to −2.57 pp; *P* < .001) to receive a response from an attending physician and 3.01 pp more likely (95% CI, 1.76-4.27 pp; *P* < .001) to receive a response from an RN (Figure 1), corresponding to a 17.4% lower attending response rate. Similar, but smaller, differences were observed for messages from Asian (−2.11 pp; 95% CI, −4.39 to 0.17 pp; *P* = .07) and Hispanic (−2.32 pp; 95% CI, −3.93 to −0.71 pp; *P* = .005) patients.

**Figure 2.**
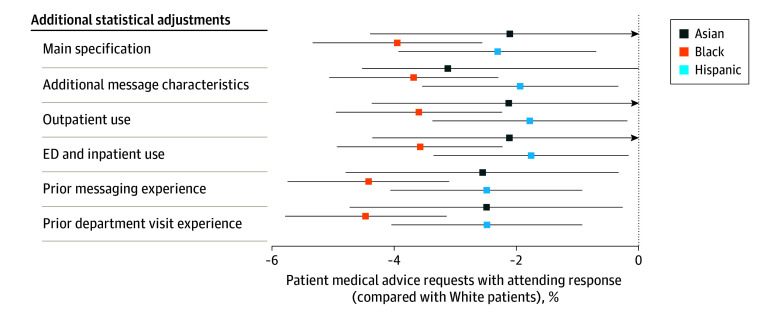
Sensitivity Analyses: Robustness of 2021 Attending Physician Response Rate Differences Each subsequent row after the first layers on additional statistical adjustments to the baseline “Main specification.” Error bars reflect 95% CIs with heteroskedasticity robust standard errors clustered at the patient level. ED indicates emergency department.

Differences in attending physician response rates were robust to additional controls for characteristics of the initiating message (whether it was sent via mobile app, the type of device that accessed the mobile app, whether it included an attachment, and the other functionalities used in the portal session in which it was sent; average message characteristics are provided in eTable 4 in Supplement 1); patients’ outpatient, ED, and inpatient care use before and after the message was sent; patients’ messaging experience in the prior year; and patients’ visit volume with the recipient department in the prior year (Figure 2). Across our subgroup sensitivity analyses, differences were largely similar, but attenuated for patients with depression diagnoses and patients with 5 or more primary care visits in 2020 (eFigure 1 in Supplement 1). Results were also similar for patients who were primary care active in 2019 vs 2020 (eFigure 2 in Supplement 1), when using random effects vs fixed effects (eFigure 3 in Supplement 1), and when examining the care team member type who sent the first response vs whether the care team member type provided any response (eFigure 4 in Supplement 1). Adjusted comparisons for additional response outcomes are provided in eTable 5 in Supplement 1.

## Discussion

The central tenet behind message triaging is that physician time is valuable and should be allocated judiciously. In our setting, physician resources—as measured by threads responded to—were disproportionately allocated toward White patients. While only 21.1% of patients in our sample were White, they received 46.3% of all attending physician responses (eTable 6 in Supplement 1).

Prior work has highlighted messaging rates as a key factor in this difference; we see that as well at BMC.^9^ However, our study also shows that, even when patients who belong to minoritized racial and ethnic groups sent messages, there were meaningful differences in the rates of attending physician response. These differences persisted even after adjusting for key factors, such as recipient department and patient health status.

For nearly all departments at BMC, medical advice request messages are sent to a department message pool with a triaging nurse. Given that we observed lower rates of attending physician response together with higher rates of RN response, we infer that the observed gap is likely the result of lower rates of message forwarding to attending physicians. These lower rates may be due to differences in the underlying message request (ie, what is the issue and what is the patient asking for), differences in the way the message was communicated (eg, specificity, tone, grammar, and spelling), or differences in the way care teams respond to the message conditional on the first 2 characteristics (eg, implicit bias). In our regression analyses we attempted to account for the first 2 facets by adjusting for a host of patient and message thread characteristics. Yet, even after these adjustments were made, differences persisted.

Future work analyzing patient portal message text will be necessary to further determine the mechanisms influencing these observed differences and understanding their ramifications. It is important to acknowledge that not all differences in care team response are necessarily of concern. No prior work that we are aware of has examined the benefits, if any, of attending physician vs nurse response to patient portal messages, and the types of messages that benefit most from attending physician response. Indeed, for many types of message requests, direct RN response may be most appropriate. However, when differences in triage prioritization are driven not by differences in the underlying message request itself, but rather by differences in the way the message is communicated or by (potentially unconscious) care team bias, we should be particularly concerned about disparities in care team resource allocation and consider potential levers to mitigate those inequities.

When gaps in attending physician response are due to differences in the underlying message requests, some of this may reflect differences in the fully informed needs and preferences of patients across race and ethnicity groups, which do not need to be addressed. However, if these differences instead reflect lower awareness among patients who belong to minoritized racial and ethnic groups of the types of requests that can be made through the patient portal, interventions aimed at improving patient portal literacy can help close the gap (eg, educational videos on portal use shown in waiting rooms, and primers and tutorials provided within the portal).

Where gaps in attending response are influenced by differences in the way in which a request is communicated, simple portal modifications—or even the application of AI (eg, support from large language models)—might help patients better categorize and structure their messages. In addition, when the gap is due to care teams, steps should be taken to counteract implicit biases, not just along patient characteristics, but message characteristics as well, such as the way grammar, vocabulary, and tone can influence perception of a message’s importance.

Since the start of the pandemic, secure portal messages have become an increasingly important channel for patient–care team interaction.^5,7^ Use of text-based health care communications is likely to continue to grow, evidenced by both traditional health care institutions, such as BMC, and the growing sector of digital-first care institutions, such as Firefly and Ginger, which often rely on text-based messaging with a health coach as a first-line triage point before patients receive clinician-based care.^22^ Additionally, while AI tools have been discussed as a means for triaging and responding to patient text-based requests,^15,16,23,24,25^ prior work has shown that AI tools can perpetuate underlying inequalities in the data that algorithms were trained on.^17,18^ Understanding and correcting existing disparities in care team response to patient messages will be vital for increasing equity in care today and the algorithms of the future.

### Limitations

Our study has a number of limitations. First, because our analysis was limited to a single health system, there may be concerns about the generalizability of our findings. Second, because of the substantial pandemic-related disruptions to outpatient care in 2020, there may be concerns that patients who were primary care active in 2020 are not representative of the general population. We aim to address these concerns through sensitivity analyses examining patients who were primary care active in 2019 rather than 2020. Third, we acknowledge that the standard race and ethnicity groups used in our study mask substantial within-group heterogeneity.^26^ Fourth, while we had access to a variety of message-level metadata characteristics, we could not observe all relevant features of patient messages. Most notably, we were not able to analyze message text itself, although we hope to in future work.

## Conclusions

In this cross-sectional study, we observed substantial differences in how care teams responded to medical advice request messages from Asian, Black, and Hispanic patients compared with messages from similar White patients. While overall care team response rates were comparable across the 4 groups, messages from patients who belong to minoritized racial and ethnic groups were far less likely to receive a response from attending physicians, suggesting lower prioritization during nurse triaging. Prior articles have noted the substantial care team resources required to address the growing volume of patient portal messages.^7,8^ Our findings highlight potential disparities in how those resources are allocated across patient groups. Understanding and addressing these disparities will be necessary for improving care equity and informing algorithms.

## Notes

Supplement 1. **eTable 1.** 27 Common Conditions Used to Represent Patient Health Status **eMethods.** Specific Methods **eTable 2.** Characteristics of Patients Who Were Primary Care Active at Boston Medical Center in 2020 and Sent at Least One Patient Medical Advice Message **eTable 3.** Additional 2021 Patient Medical Advice Request Care Team Response Outcomes for Patients Who Were Primary Care Active at Boston Medical Center in 2020 **eTable 4.** Characteristics of 2021 Patient Medical Advice Request Threads From Patients Who Were Primary Care Active at Boston Medical Center in 2020 **eFigure 1**. Subgroup Sensitivity Analysis – 2021 Attending Response Rates for Patient Medical Advice Request Threads by Patients Who Were Primary Care Active at Boston Medical Center in 2020 **eFigure 2**. Alternative Sample Sensitivity Analysis – 2021 Care Team Response Rates for Patient Medical Advice Request Threads by Patients Who Were Primary Care Active at Boston Medical Center in 2019 **eFigure 3**. Random Effects Model Sensitivity Analysis – 2021 Care Team Response Rates for Patient Medical Advice Request Threads by Patients Who Were Primary Care Active at Boston Medical Center in 2020 **eFigure 4.** 2021 First Care Team Response to Patient Medical Advice Request Threads by Patients Who Were Primary Care Active at Boston Medical Center in 2020 **eTable 5.** Additional 2021 Care Team Response Outcomes to Patient Medical Advice Request Threads by Patients Who Were Primary Care Active at Boston Medical Center in 2020 – Comparison by Patient Race and Ethnicity, Controlling for Patient Characteristics, Message Characteristics, and Recipient Department **eTable 6.** Share of 2021 Patient Medical Advice Request Threads From Patients Who Were Primary Care Active at Boston Medical Center in 2020

Supplement 2.
Data Sharing Statement
