# Supplementary material for: Differences in Care Team Response to Patient Portal Messages by Patient Race and Ethnicity
Source: JAMA Netw Open. 2024 Mar 18;7(3):e242618. doi: 10.1001/jamanetworkopen.2024.2618 (PMC10949096; doi:10.1001/jamanetworkopen.2024.2618)
Supplement: Supplement 1. — eTable 1. 27 Common Conditions Used to Represent Patient Health Status eMethods. Specific Methods eTable 2. Characteristics of Patients Who Were Primary Care Active at Boston Medical Center in 2020 and Sent at Least One Patient Medical Advice Message eTable 3. Additional 2021 Patient Medical Advice Request Care Team Response Outcomes for Patients Who Were Primary Care Active at Boston Medical Center in 2020 eTable 4. Characteristics of 2021 Patient Medical Advice Request Threads From Patients Who Were Primary Care Active at Boston Medical Center in 2020 eFigure 1. Subgroup Sensitivity Analysis – 2021 Attending Response Rates for Patient Medical Advice Request Threads by Patients Who Were Primary Care Active at Boston Medical Center in 2020 eFigure 2. Alternative Sample Sensitivity Analysis – 2021 Care Team Response Rates for Patient Medical Advice Request Threads by Patients Who Were Primary Care Active at Boston Medical Center in 2019 eFigure 3. Random Effects Model Sensitivity Analysis – 2021 Care Team Response Rates for Patient Medical Advice Request Threads by Patients Who Were Primary Care Active at Boston Medical Center in 2020 eFigure 4. 2021 First Care Team Response to Patient Medical Advice Request Threads by Patients Who Were Primary Care Active at Boston Medical Center in 2020 eTable 5. Additional 2021 Care Team Response Outcomes to Patient Medical Advice Request Threads by Patients Who Were Primary Care Active at Boston Medical Center in 2020 – Comparison by Patient Race and Ethnicity, Controlling for Patient Characteristics, Message Characteristics, and Recipient Department eTable 6. Share of 2021 Patient Medical Advice Request Threads From Patients Who Were Primary Care Active at Boston Medical Center in 2020 [file jamanetwopen-e242618-s001.pdf]

Supplementary Online Content

Tang M, Mishuris RG, Payvandi L, Stern AD. Differences in care team response to patient portal messages by patient race and ethnicity. *JAMA Netw Open*. 2024:7(3):e242618. doi:10.1001/jamanetworkopen.2024.2618

eTable 1. 27 Common Conditions Used to Represent Patient Health Status

eMethods. Specific Methods

eTable 2. Characteristics of Patients Who Were Primary Care Active at Boston Medical Center in 2020 and Sent at Least One Patient Medical Advice Message

eTable 3. Additional 2021 Patient Medical Advice Request Care Team Response Outcomes for Patients Who Were Primary Care Active at Boston Medical Center in 2020

eTable 4. Characteristics of 2021 Patient Medical Advice Request Threads From Patients Who Were Primary Care Active at Boston Medical Center in 2020

eFigure 1. Subgroup Sensitivity Analysis – 2021 Attending Response Rates for Patient Medical Advice Request Threads by Patients Who Were Primary Care Active at Boston Medical Center in 2020

eFigure 2. Alternative Sample Sensitivity Analysis – 2021 Care Team Response Rates for Patient Medical Advice Request Threads by Patients Who Were Primary Care Active at Boston Medical Center in 2019

eFigure 3. Random Effects Model Sensitivity Analysis – 2021 Care Team Response Rates for Patient Medical Advice Request Threads by Patients Who Were Primary Care Active at Boston Medical Center in 2020

eFigure 4. 2021 First Care Team Response to Patient Medical Advice Request Threads by Patients Who Were Primary Care Active at Boston Medical Center in 2020

eTable 5. Additional 2021 Care Team Response Outcomes to Patient Medical Advice Request Threads by Patients Who Were Primary Care Active at Boston Medical Center in 2020 – Comparison by Patient Race and Ethnicity, Controlling for Patient Characteristics, Message Characteristics, and Recipient Department

eTable 6. Share of 2021 Patient Medical Advice Request Threads From Patients Who Were Primary Care Active at Boston Medical Center in 2020

This supplementary material has been provided by the authors to give readers additional information about their work.

© 2024 Tang M et al. *JAMA Network Open.*

eTable 1. 27 common conditions used to represent patient health status

**Condition**
Hypertension
Hyperlipidemia
Depression
Anemia
Cataracts
Diabetes
Rheumatoid Arthritis/Osteoarthritis
Chronic Kidney Disease
Asthma
Glaucoma
Ischemic Heart Disease
Chronic Obstructive Pulmonary Disease
Acquired Hypothyroidism
Benign Prostatic Hyperplasia
Heart Failure
Stroke/Transient Ischemic Attack
Osteoporosis
Atrial Fibrillation
Acute Myocardial Infarction
Non-Alzheimer’s Dementia
Breast Cancer
Prostate Cancer
Colorectal Cancer
Lung Cancer
Alzheimer’s Disease
Hip/Pelvic Fracture
Endometrial Cancer

© 2024 Tang M et al. *JAMA Network Open.*

eMethods. Specific Methods
Ordinary Least Squares Regression Specifications

$$Y_{i,m,x,p,z,d} = \sum_{r} \beta_r R_{ir} + \beta X_i + \alpha \mathrm{M_m} + \gamma_p + \zeta_z + \delta_d + \rho_x + \epsilon_{ispzd}$$

Where *i* refers to a patient. *m* refers to a specific message thread sent to recipient department *x*. *p* refers to the patient's health insurer, *z* refers to their zip code of residence, and *d* refers to their most seen primary care provider in 2020.

$Y_{i,m,x,p,z,d}$ represents the type of response message thread (*i, m, x*) ultimately received. We examined whether the message had any care team response. We also examined the types of care team members that responded. Provider categories included: (1) attending physician, (2) registered nurse (RN), (3) advanced practice provider (APP), (4) resident physician, and (5) other (including medical assistants, social workers, etc.). Additional secondary response outcomes included (1) the number of care team responses; (2) hours until first care team response; and (3) whether the thread ended on a patient message or not. We also included a set of outcomes capturing the provider type that gave the thread's first care team response, leveraging the same provider categories noted previously.

R_{ir} refers to whether patient *i* is in race and ethnicity group *r*. The β_r values serve as our main estimates of interest, reflecting the adjusted difference in response outcomes for messages sent by patients in race and ethnicity *r*, relative to White patients, which serve as our reference group.

X_i refers to the patient's characteristics:

- Sex
- Age group: either 18-24, 25-34, 35-44, 45-54, 55-64, 65+
- Number of chronic conditions: 0, 1, 2, 3, 4, 5, 6, 7, 8+
- Whether English was their primary language

M_m refers to a message's characteristics. In our main specification we limit these to the following:

- Year-month that the message was sent
- The day of the week the message was sent
- The hour of the day the message was sent

$\gamma_p + \zeta_z + \delta_d + \rho_x$ are fixed effects for the patient's health insurer, zip code of residence, most seen primary care provider in 2020, and the recipient department the message was sent to respectively.

Standard errors were clustered at the patient level.

In robustness checks we incorporated the following groups of additional controls **(**results provided in **Figure 2)**:

© 2024 Tang M et al. *JAMA Network Open.*

1. Additional message characteristics
    - Whether the initial message included an attachment
    - Whether the initial message was sent via the mobile app
    - Whether the initial message was sent via the iPhone or Android mobile app
    - Whether the following portal functionalities were used in the same portal session that generated the initial message: “Medical Histories”, “Test Results List”, “Test Results Details”, “Medications”, “Clinical Notes”, “Upcoming Appointments”, “Visits”, “Inpatient Admissions”, “Provider List Widget”

2. Outpatient utilization
    - Whether the patient had any outpatient visits in the 14 days prior to the message, the 7 days prior to the message, the day of the message, 7 days following the message, and 14 days following the message
    - The number of PCP outpatient visits the patient had in 2020 (including both a linear and squared term)
    - The number of non-PCP outpatient visits the patient had in 2020 (including both a linear and squared term)

3. ED and inpatient utilization
    - Whether the patient had any emergency department or inpatient discharges in the 28, 21, 14, and 7 days prior to the message; the day of the message; and the 28, 21, 14, and 7 days after the message

4. Prior messaging experience
    - The number of patient medical advice request threads sent in 2020 to the specific recipient department on this message (including both a linear and squared term)
    - The number of patient medical advice request threads sent in 2020 to any department other than the recipient department (including both a linear and squared term)

5. Prior department visit experience
    - The number of outpatient visits the patient had with the recipient department in 2020 (including both a linear and squared term)

© 2024 Tang M et al. *JAMA Network Open.*

eTable 2. Characteristics of Patients Who Were Primary Care Active at Boston Medical Center in 2020 and Sent at Least One Patient Medical Advice Message

	Patient race and ethnicity group				
	**Asian**	**Black**	**Hispanic**	**White**	**Total**
Total PCP active patients, No. (% of total)	686 (6.4%)	4,793 (44.9%)	1,797 (16.8%)	3,410 (31.9%)	10,686 (100%)
Sex, No. (%)					
Female	415 (60.5%)	3,447 (71.9%)	1,276 (71.0%)	2,070 (60.7%)	7,208 (67.5%)
Male	271 (39.5%)	1,346 (28.1%)	521 (29.0%)	1,340 (39.3%)	3,478 (32.5%)
Age, Mean	42.9 (42.9)	44.3 (44.3)	43.1 (43.1)	46.9 (46.9)	44.9 (44.9)
Age category, No. (%)					
18 to 24	32 (4.7%)	211 (4.4%)	94 (5.2%)	93 (2.7%)	430 (4.0%)
25 to 34	264 (38.5%)	1,400 (29.2%)	555 (30.9%)	994 (29.1%)	3,213 (30.1%)
35 to 44	134 (19.5%)	1,066 (22.2%)	450 (25.0%)	651 (19.1%)	2,301 (21.5%)
45 to 54	86 (12.5%)	855 (17.8%)	284 (15.8%)	445 (13.0%)	1,670 (15.6%)
55 to 64	79 (11.5%)	672 (14.0%)	224 (12.5%)	615 (18.0%)	1,590 (14.9%)
65+	91 (13.3%)	589 (12.3%)	190 (10.6%)	612 (17.9%)	1,482 (13.9%)
English preferred language, No. (%)	544 (79.3%)	4,406 (91.9%)	1,271 (70.7%)	3,310 (97.1%)	9,531 (89.2%)
No. of chronic conditions, Mean	2.3	3.5	3.1	3.0	3.2
Patients with chronic conditions, No. (%)					
Hypertension	197 (28.7%)	2,284 (47.7%)	687 (38.2%)	1,280 (37.5%)	4,448 (41.6%)
Diabetes	120 (17.5%)	1,131 (23.6%)	364 (20.3%)	424 (12.4%)	2,039 (19.1%)
Hyperlipidemia	201 (29.3%)	1,476 (30.8%)	536 (29.8%)	1,091 (32.0%)	3,304 (30.9%)
Depression	153 (22.3%)	2,075 (43.3%)	849 (47.2%)	1,388 (40.7%)	4,465 (41.8%)
Arthritis	97 (14.1%)	1,201 (25.1%)	447 (24.9%)	892 (26.2%)	2,637 (24.7%)
Anemia	132 (19.2%)	1,962 (40.9%)	522 (29.0%)	669 (19.6%)	3,285 (30.7%)
Chronic kidney disease	84 (12.2%)	1,054 (22.0%)	324 (18.0%)	516 (15.1%)	1,978 (18.5%)
Cataracts	135 (19.7%)	1,074 (22.4%)	349 (19.4%)	576 (16.9%)	2,134 (20.0%)
Care utilization at BMC from 1/1/2021 through 11/24/2021, No. (Per patient average)					

© 2024 Tang M et al. *JAMA Network Open.*

Primary care visits	1,524 (2.22)	15,040 (3.14)	5,808 (3.23)	8,008 (2.35)	30,380 (2.84)
Non-primary care visits	6,127 (8.93)	56,234 (11.73)	21,893 (12.18)	37,269 (10.93)	121,523 (11.37)
Emergency department visits	107 (0.16)	2,548 (0.53)	903 (0.50)	726 (0.21)	4,284 (0.40)
Inpatient admissions	46 (0.07)	613 (0.13)	199 (0.11)	321 (0.09)	1,179 (0.11)
Zip code income quintile, No. (%)					
Highest income quintile	347 (50.6%)	1,671 (34.9%)	642 (35.7%)	2,067 (60.6%)	4,727 (44.2%)
2nd income quintile	215 (31.3%)	829 (17.3%)	484 (26.9%)	818 (24.0%)	2,346 (22.0%)
3rd income quintile	88 (12.8%)	1,275 (26.6%)	313 (17.4%)	299 (8.8%)	1,975 (18.5%)
4th income quintile	12 (1.7%)	183 (3.8%)	109 (6.1%)	97 (2.8%)	401 (3.8%)
Lowest income quintile	20 (2.9%)	821 (17.1%)	244 (13.6%)	97 (2.8%)	1,182 (11.1%)

Notes: Table shows characteristics for patients who were primary care active at BMC in 2020 (at least 1 primary care outpatient visit in 2020) and had sent at least one patient medical advice request message in 2021. Patient race and ethnicity groups were determined based on patient self-reported data recorded in BMC's electronic health record. All patients of Hispanic or Latino ethnicity were grouped together into the "Hispanic" group, regardless of race. The remaining non-Hispanic patients were categorized by race. Patients with a recorded race of "Native Hawaiian or Other Pacific Islander", "American Indian or Alaska Native", or "Other" were excluded due to insufficient sample size. Patients with unknown race and ethnicity were excluded as well. Patient age, zip code of residence, and health status reflected their latest record as of 11/24/21 (end of our study period). Patients' health status was represented through binary indicators for 27 common conditions (full list provided in eTable 1). These indicators were populated based on patients' EHR records, including their outpatient encounter diagnoses, diagnoses on their "problem list", and primary diagnoses from their inpatient encounters. "Zip code income quintiles" are based on data from the 2022 American Community Survey and represent the median household income for patients' zip code of residence. 0.5% of patients had missing zip code of residence data and were not included in the zip code income quintile measures.

© 2024 Tang M et al. *JAMA Network Open.*

eTable 3. Additional 2021 Patient Medical Advice Request Care Team Response Outcomes for Patients Who Were Primary Care Active at Boston Medical Center in 2020

	Patient race and ethnicity group				
	**Asian**	**Black**	**Hispanic**	**White**	**Total**
Total PCP active patients, No. (% of total)	2,006 (5.1%)	21,600 (55.3%)	7,185 (18.4%)	8,252 (21.1%)	39,043 (100%)
Patient medical advice request threads, No. (% of total)	2,714 (4.7%)	23,174 (40.2%)	8,804 (15.3%)	23,012 (39.9%)	57,704 (100%)
Patient medical advice request threads - first response, No. (% of threads sent by group)					
Attending physician	539 (19.9%)	3,164 (13.7%)	1,335 (15.2%)	4,440 (19.3%)	9,478 (16.4%)
Registered nurse	838 (30.9%)	7,603 (32.8%)	2,738 (31.1%)	6,068 (26.4%)	17,247 (29.9%)
Advanced practice provider	128 (4.7%)	1,445 (6.2%)	578 (6.6%)	1,477 (6.4%)	3,628 (6.3%)
Resident	124 (4.6%)	528 (2.3%)	203 (2.3%)	483 (2.1%)	1,338 (2.3%)
Other	294 (10.8%)	2,632 (11.4%)	1,010 (11.5%)	2,308 (10.0%)	6,244 (10.8%)
Number of care team responses to patient medical advice request threads, No. (Mean per thread)	2,884 (1.06)	22,710 (0.98)	8,520 (0.97)	22,206 (0.96)	56,320 (0.98)
Patient medical advice request threads hours to first response, Median	15.4	13.3	12.8	13.6	13.5
Patient medical advice threads with final message sent by patient, No. (% of threads sent by group)	1,174 (43.3%)	11,371 (49.1%)	4,171 (47.4%)	11,766 (51.1%)	28,482 (49.4%)

Notes: Table shows additional response outcomes for medical advice requests sent from 1/1/2021 through 11/24/2021 by Asian, Black, Hispanic, or White patients who were primary care active at BMC in 2020 and is aimed to complement the primary outcomes examined in Table 2 in the main text. Medical advice request threads include a patient-initiated initial message and all other messages in the response chain.

© 2024 Tang M et al. *JAMA Network Open.*

eTable 4. Characteristics of 2021 Patient Medical Advice Request Threads From Patients Who Were Primary Care Active at Boston Medical Center in 2020

	Patient race and ethnicity group				
	**Asian**	**Black**	**Hispanic**	**White**	**Total**
Patient medical advice request threads, No.	2,714	23,174	8,804	23,012	57,704
Patient medical advice request thread characteristics, No. (% of threads)					
Initial message has attachment	283 (10.4%)	2,141 (9.2%)	918 (10.4%)	1,972 (8.6%)	5,314 (9.2%)
Initial message sent outside working hours	927 (34.2%)	7,521 (32.5%)	2,903 (33.0%)	7,307 (31.8%)	18,658 (32.3%)
Initial message sent via mobile app	1,213 (44.7%)	14,171 (61.2%)	6,049 (68.7%)	10,333 (44.9%)	31,766 (55.0%)
Initial message sent via Android mobile app	246 (9.1%)	5,057 (21.8%)	1,774 (20.1%)	3,810 (16.6%)	10,887 (18.9%)
Initial message sent via iPhone mobile app	967 (35.6%)	9,114 (39.3%)	4,275 (48.6%)	6,523 (28.3%)	20,879 (36.2%)
Portal function was accessed in same session as initial message, No. (% of threads)					
Medical Histories	924 (34.0%)	8,603 (37.1%)	3,626 (41.2%)	7,398 (32.1%)	20,551 (35.6%)
Test Results Details	317 (11.7%)	3,176 (13.7%)	1,228 (13.9%)	2,453 (10.7%)	7,174 (12.4%)
Upcoming Appointment Details	281 (10.4%)	3,034 (13.1%)	1,222 (13.9%)	2,762 (12.0%)	7,299 (12.6%)
Medications	344 (12.7%)	3,154 (13.6%)	1,219 (13.8%)	3,566 (15.5%)	8,283 (14.4%)
View Clinical Notes	152 (5.6%)	1,427 (6.2%)	588 (6.7%)	1,343 (5.8%)	3,510 (6.1%)
Number of patient advice request messages, No. (Mean per thread)	4,085 (1.51)	34,519 (1.49)	12,688 (1.44)	34,381 (1.49)	85,673 (1.48)

Notes: Table shows characteristics of medical advice requests sent from 1/1/2021 through 11/24/2021 by Asian, Black, Hispanic, or White patients who were primary care active at BMC in 2020. Medical advice request threads include a patient-initiated initial message and all other messages in the response chain; however, all characteristics shown here focus on the initial message in the thread, with the exception to the last row. Each thread was linked to the portal session in which the initial message was sent. For each portal session we observed whether the session was conducted via Epic's MyChart mobile app or through a web browser, and which type of mobile device was used to access the mobile app (Android of iPhone). Additionally, we observed which portal functionalities were accessed during the portal sessions (e.g., Messaging, Medications, Clinical Notes, etc.).

© 2024 Tang M et al. *JAMA Network Open.*

eFigure 1. Subgroup Sensitivity Analysis – 2021 Attending Response Rates for Patient Medical Advice Request Threads by Patients Who Were Primary Care Active at Boston Medical Center in 2020

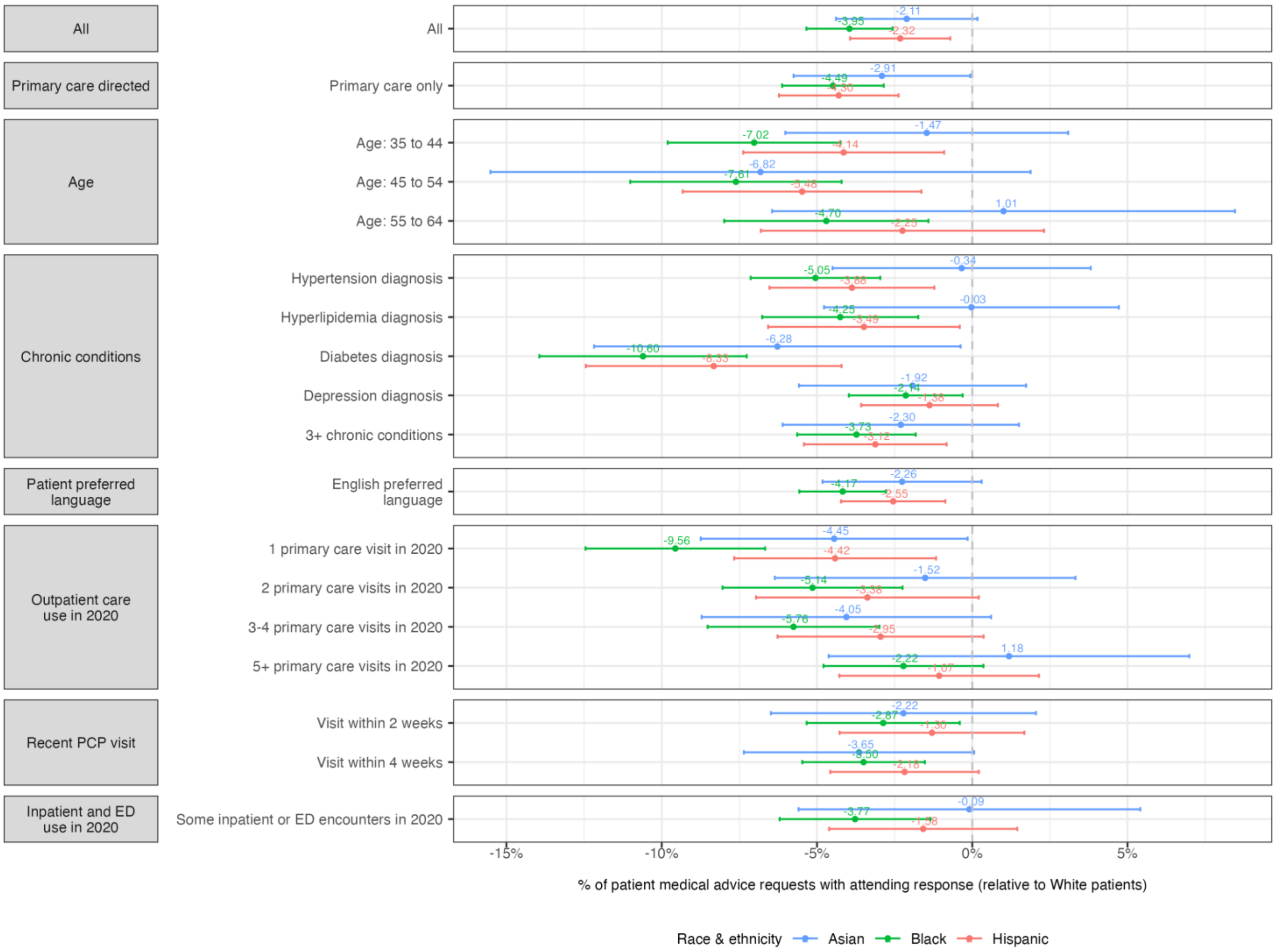

© 2024 Tang M et al. *JAMA Network Open.*

Notes: Each row displays the regression coefficients for a specific patient subgroup, with the exception of the “Primary care only” row which corresponds to message threads sent to primary care departments. Analysis was limited to patient-initiated medical advice requests sent from 1/1/2021 through 11/24/2021 by Asian, Black, Hispanic, or White patients that were primary care active in 2020 (at least 1 primary care outpatient visit in 2020). Results were adjusted for patient characteristics including sex, age, number of chronic conditions, and whether English was their preferred language. Results were also adjusted for the date and time the message was initially sent. Fixed effects were included for the patient’s health insurer, zip code of residence, most seen primary care provider in 2020, and the recipient department. Error bars reflect 95% confidence intervals with heteroskedasticity robust standard errors clustered at the patient level.

© 2024 Tang M et al. *JAMA Network Open.*

eFigure 2. Alternative Sample Sensitivity Analysis – 2021 Care Team Response Rates for Patient Medical Advice Request Threads by Patients Who Were Primary Care Active at Boston Medical Center in 2019

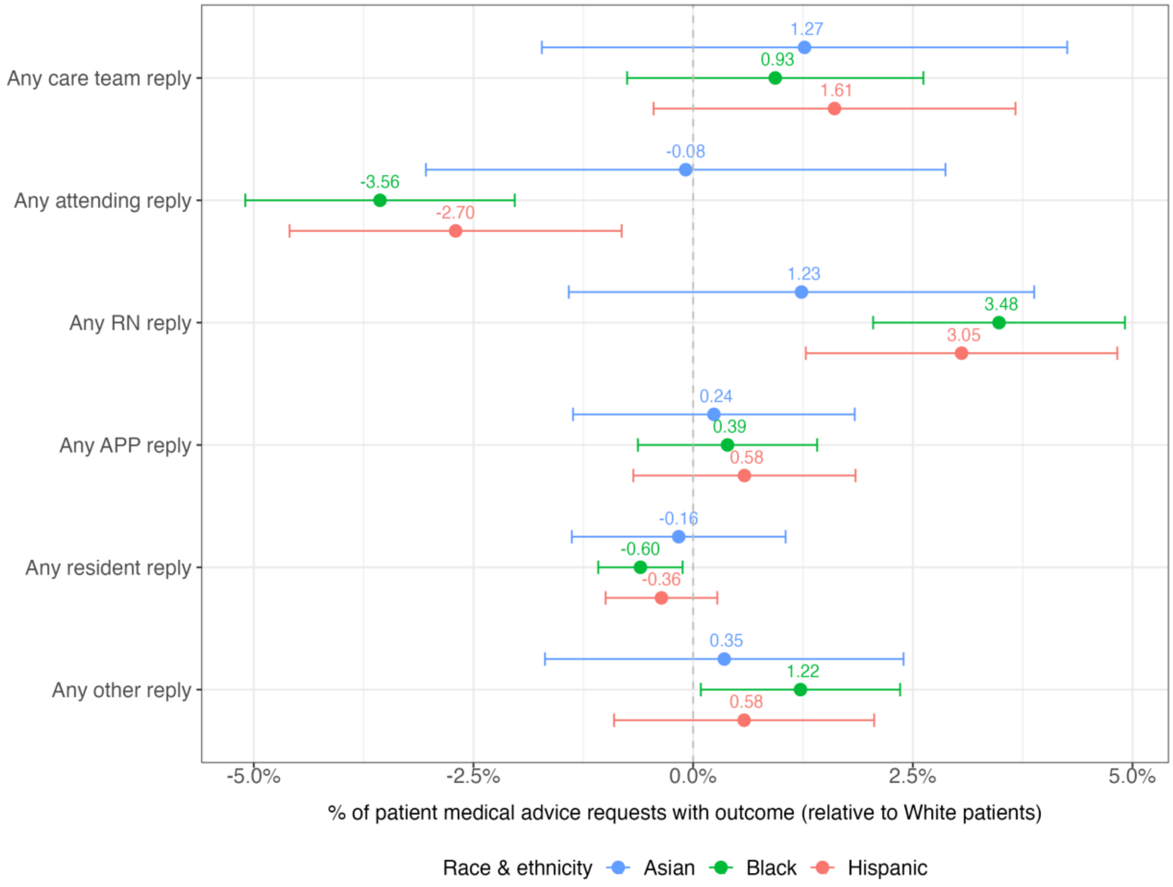

Notes: These estimates show robustness of our findings to an alternative sample definition examining 2021 medical advice requests from patients that were primary care active in 2019, rather than 2020. This alternative approach avoids overlap between our patient sample qualification period and early disruptions from the pandemic. Similar to our main specification, results were adjusted for patient characteristics including sex, age, number of chronic conditions, and whether English was their preferred language. Results were also adjusted for the year-month, the day of the week, and the hour of the day the message was initially sent. Fixed effects were included for the patient's health insurer, zip code of residence, most seen primary care provider in 2019, and the recipient department. Error bars reflect 95% confidence intervals with heteroskedasticity robust standard errors clustered at the patient level.

© 2024 Tang M et al. *JAMA Network Open.*

eFigure 3. Random Effects Model Sensitivity Analysis – 2021 Care Team Response Rates for Patient Medical Advice Request Threads by Patients Who Were Primary Care Active at Boston Medical Center in 2020

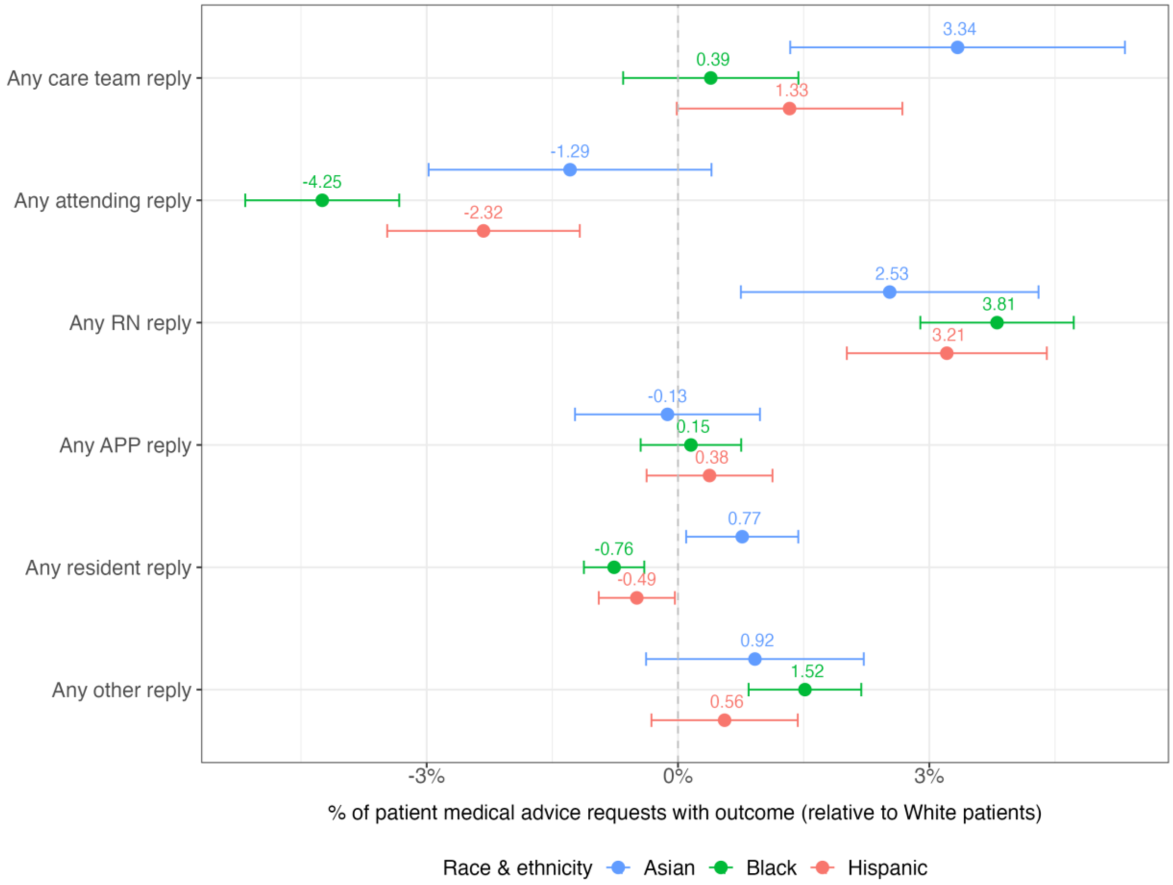

Notes: These estimates show robustness of our findings to an alternative regression specification that uses random, rather than fixed effects, to adjust for the threads' recipient department and patients' health insurer, zip code of residence, and most seen primary care provider in 2020. Analysis was limited to patient-initiated medical advice requests sent from 1/1/2021 through 11/24/2021 by Asian, Black, Hispanic, or White patients that were primary care active in 2020 (at least 1 primary care outpatient visit in 2020). Similar to our main specification, results were adjusted for patient characteristics including sex, age, number of chronic conditions, and whether English was their preferred language. Results were also adjusted for the year-month, the day of the week, and the hour of the day the message was initially sent. Error bars reflect 95% confidence intervals.

© 2024 Tang M et al. *JAMA Network Open.*

eFigure 4. 2021 First Care Team Response to Patient Medical Advice Request Threads by Patients Who Were Primary Care Active at Boston Medical Center in 2020

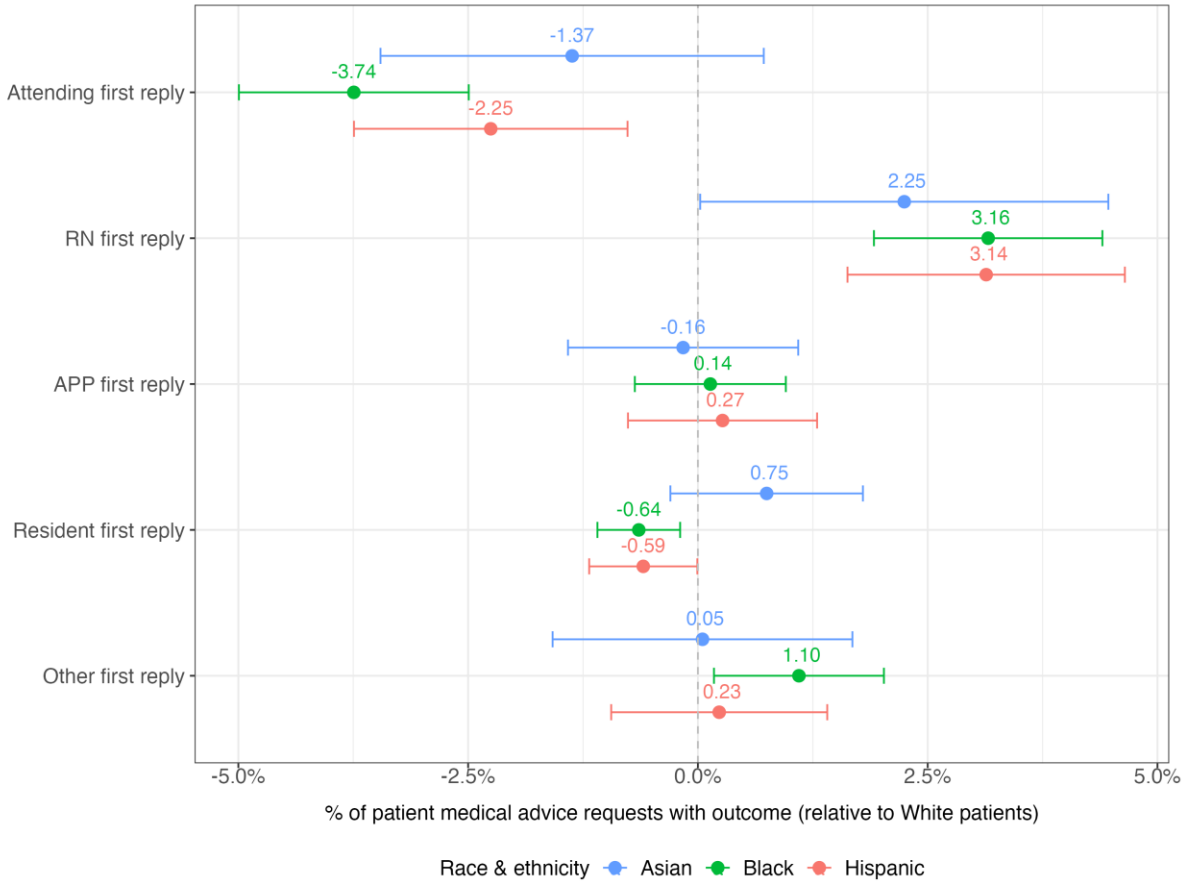

Notes: This figure shows results for a secondary set of care response outcomes that capture the provider type that gave the thread's first care team response (rather than examining whether that provider type provided any response over the course of the thread). Analysis was limited to patient-initiated medical advice requests sent from 1/1/2021 through 11/24/2021 by Asian, Black, Hispanic, or White patients that were primary care active in 2020 (at least 1 primary care outpatient visit in 2020). Results were adjusted for patient characteristics including sex, age, number of chronic conditions, and whether English was their preferred language. Results were also adjusted for the year-month, the day of the week, and the hour of the day the message was initially sent. Fixed effects were included for the patient's health insurer, zip code of residence, most seen primary care provider in 2020, and the recipient department. Error bars reflect 95% confidence intervals with heteroskedasticity robust standard errors clustered at the patient level

© 2024 Tang M et al. *JAMA Network Open.*

eTable 5. Additional 2021 Care Team Response Outcomes to Patient Medical Advice Request Threads by Patients Who Were Primary Care Active at Boston Medical Center in 2020 – Comparison by Patient Race and Ethnicity, Controlling for Patient Characteristics, Message Characteristics, and Recipient Department

Outcome	Coefficient (95% CI)	P value
Number of care team responses		
Asian	-0.011 (-0.072 to 0.051)	0.734
Black	-0.03 (-0.061 to 0.001)	0.056
Hispanic	-0.055 (-0.093 to -0.016)	0.006
Hours to first reply		
Asian	3.87 (-1.18 to 8.93)	0.133
Black	-1.79 (-4.78 to 1.21)	0.242
Hispanic	0.04 (-3.58 to 3.65)	0.984
Percent of threads with final message sent by patient		
Asian	-0.035 (-0.063 to -0.007)	0.015
Black	-0.011 (-0.03 to 0.008)	0.248
Hispanic	-0.021 (-0.042 to -0.001)	0.045

Notes: This table shows regression coefficients for additional secondary response outcomes. Analysis was limited to patient-initiated medical advice requests sent from 1/1/2021 through 11/24/2021 by Asian, Black, Hispanic, or White patients that were primary care active in 2020 (at least 1 primary care outpatient visit in 2020). Results were adjusted for patient characteristics including sex, age, number of chronic conditions, and whether English was their preferred language. Results were also adjusted for the year-month, the day of the week, and the hour of the day the message was initially sent. Fixed effects were included for the patient's health insurer, zip code of residence, most seen primary care provider in 2020, and the recipient department. Error bars reflect 95% confidence intervals with heteroskedasticity robust standard errors clustered at the patient level

© 2024 Tang M et al. *JAMA Network Open.*

eTable 6. Share of 2021 Patient Medical Advice Request Threads From Patients Who Were Primary Care Active at Boston Medical Center in 2020

	Patient race and ethnicity group				
	**Asian**	**Black**	**Hispanic**	**White**	**Total**
Total PCP active patients, No. (% of total)	2,006 (5.1%)	21,600 (55.3%)	7,185 (18.4%)	8,252 (21.1%)	39,043 (100%)
Patient medical advice request threads, No. (% of total)	2,714 (4.7%)	23,174 (40.2%)	8,804 (15.3%)	23,012 (39.9%)	57,704 (100%)
Patient medical advice request threads with care team response, No. (% of total)					
Any care team response	1,923 (5.1%)	15,372 (40.5%)	5,864 (15.5%)	14,776 (39.0%)	37,935 (100%)
Any attending physician response	601 (5.3%)	3,861 (34.2%)	1,592 (14.1%)	5,220 (46.3%)	11,274 (100%)
Any registered nurse response	872 (4.9%)	7,897 (44.0%)	2,832 (15.8%)	6,335 (35.3%)	17,936 (100%)
Any advance practice provider response	150 (3.6%)	1,657 (40.2%)	667 (16.2%)	1,646 (40.0%)	4,120 (100%)
Any resident response	131 (8.8%)	572 (38.2%)	241 (16.1%)	553 (36.9%)	1,497 (100%)
Any other response	338 (4.9%)	2,901 (41.9%)	1,110 (16.0%)	2,576 (37.2%)	6,925 (100%)

Notes: This table examines the share of medical advice threads with various response outcomes accounted for by each patient race and ethnicity group (e.g., White patients accounted for 46.3% of all medical advice threads that received an attending physician response). Analysis was limited to patient-initiated medical advice requests sent from 1/1/2021 through 11/24/2021 by Asian, Black, Hispanic, or White patients that were primary care active in 2020 (at least 1 primary care outpatient visit in 2020).

© 2024 Tang M et al. *JAMA Network Open.*
