# Supplementary material for: Differences in Care Team Response to Patient Portal Messages by Patient Race and Ethnicity
Source: JAMA Netw Open. 2024 Mar 18;7(3):e242618. doi: 10.1001/jamanetworkopen.2024.2618 (PMC10949096; doi:10.1001/jamanetworkopen.2024.2618)
Supplement: Supplement 2. — Data Sharing Statement [file jamanetwopen-e242618-s002.pdf]

Data Sharing Statement

Tang. Differences in Care Team Response to Patient Portal Messages by Patient Race and Ethnicity. *JAMA Netw Open*. Published March 18, 2024.
doi:10.1001/jamanetworkopen.2024.2618

Data
Data available: No

Additional Information
Explanation for why data not available: These data are highly confidential and require access via Boston Medical Center. We are happy to make analysis code available to any interested researchers.
